# COVID-19 Pandemic and Microplastic Pollution

**DOI:** 10.3390/nano12050851

**Published:** 2022-03-03

**Authors:** Minha Lee, Heejung Kim

**Affiliations:** Department of Geology, Kangwon National University, Chuncheon 200-701, Korea; minha_lee@korea.ac.kr

**Keywords:** COVID-19 pandemic, microplastic, personal protective equipment, recycle, waste management

## Abstract

The world is suffering from aggravating, waste-generated consequences, and the contribution of microplastics to this problem is only increasing. A contributing factor to increased microplastic usage is the change in the use of personal protective equipment (PPE) from specific use in limited locations (e.g., hospitals) to general use in widespread locations to protect against the current COVID-19 pandemic. This has resulted in an overflow of microfiber waste from homes, schools, streets, and elsewhere, in every country. While various institutes have issued warnings regarding increasing PPE waste, there is no positive indication of an end to the pandemic in the near future. In this review, we examine the impact of the pandemic on microplastic production, consumption, and disposal, and suggest strategies for lessening environmental pollution. In preparation for the worst-case scenario in which PPE becomes a new normal (in the COVID-19 era), it is recommended that governments and other responsible organisations set up a structured monitoring system for the distribution and disposal of PPE to ensure the most effective waste management possible for continuous sustainable development.

## 1. Introduction

Personal protective equipment (PPE) (i.e., masks, face shields, gowns, and gloves) has become a major source of microplastics pollution, especially as environmental pollutants to aquatic systems. In pre-COVID-19 times, PPE was mainly used in a controlled manner typically by healthcare service providers and at research laboratories. Thus, the handling and disposal procedures were managed by trained specialists under the protocols specified by related governmental laws and regulations [1,2]. However, with the COVID-19 pandemic, PPE is being used and disposed of widely. Almost every individual, including young toddlers above age 2 years, uses PPE daily under the mandated or recommended facial covering measures aimed at mitigating the spread of severe acute respiratory syndrome coronavirus 2 (SARS-CoV-2). However, disposal control has lagged relative to the surging demand.

As the daily use of PPE by the public became the norm, the frequency of spotting littered face masks and gloves increased to the level of several units per day. National Geographic reported that the “discarded PPE has clogged street drains from New York City to Nairobi and has gummed up machinery in the municipal sewage system in Vancouver, British Columbia” [3]. The problem lies in the fact that PPE is neither recyclable in most municipal systems, nor is it biodegradable. Masks typically comprise a mix of paper and polymers, including polypropylene, commonly used in surgical masks and, as a result, masks have a high potential to release massive quantities of microfibres into the sea [4]. Considering these circumstances, we reviewed the recent literature to shed light on the increased threat of microplastic pollution to sustainable development in the post-COVID-19 world. We aimed to outline analysis-based policy recommendations to control the release of microplastics in the natural environment in light of the unavoidable use of PPE to fight the spread of SARS-CoV-2 and other viruses.

## 2. COVID-19 Pandemic Shock on Plastic Production and Consumption

Global plastic production has increased from 1.5 million metric tonnes in 1950, to 250 million metric tonnes in 2009, and 368 million tonnes in 2019 [5]. To add another burden, the world is experiencing a pandemic in which PPE is in great demand for health and safety purposes. At the outbreak of the COVID-19 pandemic, the World Health Organization (WHO) encouraged the global market to expedite PPE production by 40% monthly to effectively suppress the rapid spread of SARS-CoV-2 [6]. The production volume reached 2,452 billion pieces in 2020, while the value of EU imports of face masks grew from EUR 800 million in the first term of 2019 to EUR 14 billion for the same period in 2020 [7]. In China, 200 million face masks of various types were produced per day as of June 2020, a 20-fold increase in production volume since the beginning of 2020 [8]. The number of manufacturers also increased exponentially from 137 companies in January 2020 to 1,615 in September 2021 in South Korea alone [9]. The market size of two of the most immediately needed products to combat the pandemic, facial masks and disease test kits, expanded exponentially compared to the pre-pandemic period in 2019 (Table 1). According to the National Geographic Environment Newsletter published on 15 April 2021, “globally, 65 billion gloves are used every month. The tally for face masks is nearly twice that—129 billion a month. That translates into 3 million face masks used per minute. It is also reported that 3.4 billion face masks or face shields are discarded every day” [3].

**Figure 1:**
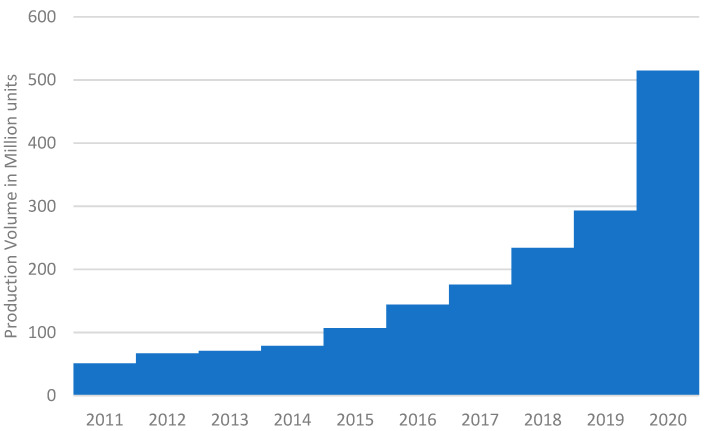
Increasing antibacterial wet wipe production in Japan (2011–2020). Source: Statista Data (https://www.statista.com/statistics/) (accessed on 19 February 2022).

PPE is not the only source of increased plastic consumption during the pandemic. The consequent lockdowns altered nearly everyone’s daily routine and personal preferences; even those who were environmentally alert and previously shopped with their own reusable eco-friendly bags leaned toward deliveries and individual packaging in fear of COVID-19 [10]. The new COVID-19 protocols, which included disinfecting and hand sanitising after each turn (e.g., wiping the cashier’s platform after each customer) drove the surging demand for disposable bottles of hand sanitiser and disinfectant wipes. A recent survey conducted in South Korea revealed that 54% of respondents began using hand sanitiser after the COVID-19 outbreak [9]. The volume of antibacterial wipe production, the largest segment of the wet wipe production in Japan, recorded a decade high increase rate of 76% in 2020 (25% in 2019) as an aftermath of the COVID-19 protocols (Figure 1, Statista Data). The Thailand Environment Institute [11] reported that Thailand consumed around 62% more plastics in April 2020 than in the past 12 months. The World Economic Forum (WEF in Davos-Klosters, Switzerland, 2020) anticipated the growing trend in e-commerce and takeaway services, which inevitably boosted further increases in plastic production and usage by 14% in the USA and 40% in Spain [12]. Business Insider (2020) also estimated that the plastic packaging market would grow “from USD 909.2 billion in 2019 to USD 1012.6 billion in 2021 with the annual growth rate of 5.5%” due to a panic-driven consumption trend during the pandemic [13]. The food delivery transaction volume in South Korea doubled in 2020 (USD 14.5 billion) from 2019 and triggered an increase in plastic food container production by 20% (110,000 tonnes) [14].

There were two other factors that drove the surge in plastic consumption during the pandemic: one was the paused regulations on the use of plastics in fear of higher virus spread at the local level [15] and the petroleum price drop due to reduced oil demand at the global level [16]. Canada, Portugal, the U.K., and the USA were among many other countries who lifted various bans on single-use plastics temporarily due to the higher risk of virus transmission. Furthermore, the oil price reduction translated into a plastic production cost reduction, of which industries took advantage f to circumvent plastic recycling. The price pressure from each government and the quality control mandate on COVID-19-related items (such as masks, single-use testing kits, and vaccination syringes) promoted the production of virgin plastics from crude oil for a higher quality at cheaper price.

## 3. Microplastic Pollution and Related Policies

### 3.1. Microplastic Pollution in Water, Soil, Air, and Food Chains

Microplastics, according to the Britannica dictionary, are plastics less than 5 mm in length, among which the primary type is present in a variety of products (e.g., microbeads, tiny pellets, and plastic fibres) and the secondary type forms in nature as larger plastics degrade. A good example of primary microplastics is the exfoliants in personal care products (e.g., soaps, sanitisers, and toothpastes), which are usually synthetic polymers [17]. Synthetic polymers enter the aquatic ecosystem by easily evading the sewage system. A research study from Plymouth University, U.K. reported that a 150 mL tube of facial scrub contains around 2.8 million microbeads, and 6 kg of acrylic clothing (e.g., fleece jackets and sportswear) emits more than 700,000 microfilaments in one cycle of a washing machine [18]. Larger plastics, either landfilled or mismanaged, biologically degrade [19,20]. Recent studies have reported that the degradation of larger plastics, including that of biodegradable polymers, occurs more rapidly than previously thought [21,22]. The presence of leachate proves moisture in landfills and imperfect closure leaves room for the infiltration of precipitation and other liquids into the waste mass.

**Figure 2:**
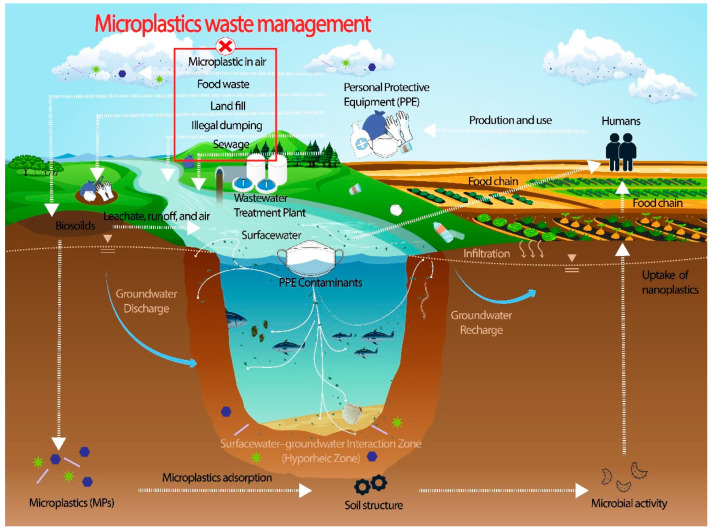
Microplastic-tailored waste management system development is urgently required to address the threats of microplastic pollution.

Plastics, when degraded or present as microplastics in the natural environment, release toxic chemicals including phthalates and organotin compounds [7]. There are five known routes in which human plastic products reach the natural ecosystem in the form of microplastics: (1) through decomposition of solid waste into soil (landfill); (2) through sludge from wastewater treatment plants (mainly rinse-off products); (3) through plastics embedded in food waste (plastics absorbed into the food chain and residuals from food packaging); (4) from illegal dumping of plastic wastes directly into nature (e.g., beaches, mountains, and lakes) [4,17,21]; and (5) microplastic particles directly released into the air [23,24] (summarised in Figure 2).

While the microbeads from rinse-off personal care products or microfibres from laundry directly enter the aquatic system, mismanaged plastic water bottles and facial masks can contribute to aqueous microplastic concentration through degradation. As food production is typically located close to water sources (i.e., lakes, rivers, and oceans), the risk of these toxic chemicals entering the food chain through plant uptake of nanoplastics is very high [20,25]. Such interaction can alter soil structure through microplastic adsorption and trigger microbial activities [26]. Furthermore, once microplastics reach the ocean, the consequences may impact entire terrestrial ecosystems. The physical appearance and odour (especially the sulphur odour of dimethyl sulphide) of microplastics can lead to them being easily mistaken for krill and other micro-sized food by fish and birds [27]. This may cause early death of wildlife and other diseases in nature, which are easily transmitted up the food chain to reach humans [28]. In addition, the microplastics generated from laundry dryer cycles, abrasion of rubber tires, and construction dusts are released directly into the air, greatly contributing to particulate matter air pollution. COVID-19 adds another burden to this air pollution, as the nonwoven layer of facial masks releases around 100 microplastics when unused and up to 1500 when in use, during which breathing action acts as an air pump [29].

### 3.2. Current Microplastic Pollution Reduction Policies

The risks of secondary microplastics were traditionally thought to be low, as plastic products were believed to be too stable to degrade naturally; thus, the only burden was to find more space (i.e., in landfills) to bury them. So far, no tailor-made waste management system has been developed for plastics, other than recycling. Countries have been burying plastics with other solid waste in landfills, and sewage systems lack a proper filtration system against microplastics [30]. However, recent studies confirm that landfill is not a suitable waste management policy for plastics and recycling is not sufficient to address rapidly accumulating plastic wastes. The United States Environmental Protection Agency (EPA) reported 35.7 million tonnes of plastic generation in the USA, accounting for 12.2% of total municipal solid waste. Only 8.7% of this plastic waste was recycled according to the Association of Plastic Recyclers of the U.S., excluding nondurable products such as disposable diapers, trash bags, utensils, and medical devices. The highest recycling rate was observed for high-density polyethylene (HDPE) natural bottles (29.3% in 2018), closely followed by that of polyethylene terephthalate (PET) bottles and jars (29.1% in 2018). The USA combusted 5.6 million tonnes of plastics (16.3% of all municipal solid waste) with energy recovery in 2018 [31].

Since there is no practical, scientific way to expunge microplastics from nature, the best solution is to prevent the initial release. To implement this approach, many countries have adopted legal restrictions prohibiting the use of microplastics in various items [32]. The Microbead-Free Waters Act of the U.S., signed in December 2015, was the first national regulation addressing microplastics: it bans the manufacture and distribution of rinse-off cosmetics products that contain plastic microbeads. It was soon expanded to non-prescription drugs but excludes all other medicines. France has exempted biodegradable alternatives of natural origins, and Sweden allows natural plastic polymers in cosmetics. The countries with maximum coverage on their legal restrictions currently are Italy, South Korea, and the U.K. [20]. In 2019, 170 nations pledged to “significantly reduce use of plastic by 2030”. Among many other countries, Kenya banned single-use plastic bags in 2017 and prohibited tourists from using single-use plastics such as water bottles and disposable plates in national parks, forests, beaches, and conservation areas since 2019 [12]. The EU Directive 2015/720, the amendment of Directive 94/62/EC about reducing the lightweight plastic carrier bags use, was effectuated as a response to growing concerns about microplastics entering the global food chain through soil and aquatic pollution and the decomposition of larger plastic products in landfills or leakage to rivers and oceans. The European Parliament further expanded the restriction to all single-use plastics, including straws, by 2021 under the “Single-use Plastics Directive” [33].

These restrictive plastic policies previously implemented in alignment with the United Nations “war on plastics” in the early 2000s, however, have been greatly disrupted by the COVID-19 pandemic. Many have been halted [34,35], and some interest parties are seizing this opportunity to re-establish plastic use for various reasons including sanitary and safety. As a result, the “ban on banning” legislation to prevent local governments from adopting environmental measures against single-use plastics and to protect the local plastic related industries in 17 states of the USA [36] is gaining more social acceptance. Moreover, there is yet to be a distinctive waste management protocol issued for general PPE use outside professionally managed sites such as hospitals and research laboratories. The only viable effort seems to be public campaigns against illegal dumping. Currently, the global standard for individual PPE waste management is to dispose of it with other general waste as it is not subject to recycling. Despite the increasing number of home-treated COVID-19 patients worldwide due to hospital capacity deficiency, there have been no separate guidelines issued for contaminated PPE waste which is entering landfill unguarded as general waste. This practice clearly leaves a critical door opened for virus transmission from microplastic waste, especially when mismanaged.

## 4. Conclusions

An increasing number of publications is warning about the danger of microplastics in the natural environment. Biodegradation of plastics takes much longer than the life span of any living organism, including humans; thus, the invasion of microplastics in the food chain is a threat to the sustainable development of civilisation. Solution-seeking should always begin with admitting the facts: many types of plastics are currently indispensable and irreplaceable, and science and technology have yet to devise a safe way to dispose of plastics. The world needs PPE to combat novel viruses, matter-borne air pollution, workplace dangers, and to practice medicine safely.

Unfortunately, landfilling, a traditional plastic waste management method, is no longer an answer and recycling, a conventional environmental policy to address plastic pollution, is insufficient to deal with the rapidly increasing plastic waste volume. Incineration, which is known for its toxic byproducts, is even more inadvisable due to its direct release of particulate matter into the air. Large plastics are degrading at a faster rate than previously thought and microplastics have been used generously in a wide range of products, making microplastics ubiquitous in water, soil, air, and food chains. Biodegradable plastics are also found to be a source of secondary microplastic pollution, and the new mega source of plastic demand, PPE, poses the health risk of possible virus transmission.

The world needs a new plastic management strategy. A separate waste protocol should be carefully designed for individual PPE use, which, by definition, is not ordinary household general waste. Facial masks, testing kits, and gloves should be treated as medical waste, especially the contaminated ones used by home-treated COVID-19 patients, like other medical wastes from hospitals and research laboratories for health and safety issues. A thorough investigation on the residual amount of PPE waste from home patients and the likelihood of virus transmission through microplastics in the ecosystem (with emphasis on the food chain accumulation) may offer a good starting point for such guideline planning.

Regarding non-PPE plastics, policies and regulations need to be re-implemented to reduce the use of plastics that are dispensable and replaceable. For recycling to be considered as an effective plastic pollution policy, public awareness on how to recycle types of plastics needs to be greatly enhanced through education. Lastly, governments should also adopt the latest technologies such as microorganisms that can biodegrade plastics in sewage wastewater and other contaminated environments to lessen existing microplastic pollution. The ready application of new technologies and materials at the national level will encourage further research and development in the relevant fields and lead to improvements.

## Figures and Tables

**Table 1:** Changed market sizes in South Korea during the pandemic (unit: billion USD).

Facial Masks			Disease Test Kits		
2019	2020	Increase	2019	2020	Increase
187	1713	816%	45	1193	2551%

Source: Ministry of Food and Drug Safety of South Korea (https://www.mfds.go.kr/eng/index.do) (accessed on 18 October 2021).

## Data Availability

Publicly available datasets were analyzed in this study. This data can be found here: [https://www.statista.com; http://plasticnet.kr; https://www.epa.gov/facts-and-figures-about-materials-waste-and-recycling/plastics-material-specific-data].
